# Supplementary material for: The Impact of Prebiotic, Probiotic, and Synbiotic Supplements and Yogurt Consumption on the Risk of Colorectal Neoplasia among Adults: A Systematic Review
Source: Nutrients. 2022 Nov 21;14(22):4937. doi: 10.3390/nu14224937 (PMC9697560; doi:10.3390/nu14224937)
Supplement: Supplementary file 1 [file nutrients-14-04937-s001.zip › nutrients-2019966-supplementary.pdf]

Supplementary Material 1- Search Terms

Search Terms

PubMed – Search performed 30 April, 692 results // Search performed May 12, 833 results

Core Search:

((((((((((((((((("Colorectal Neoplasms"[Mesh]) OR "Colorectal Neoplasms"[Majr]) OR "Colonic Neoplasms"[Mesh:NoExp]) OR "Colonic Neoplasms"[Majr:NoExp]) OR "Sigmoid Neoplasms"[Mesh:NoExp]) OR "Sigmoid Neoplasms"[Majr:NoExp]) OR "Adenomatous Polyposis Coli"[Mesh:NoExp]) OR "Adenomatous Polyposis Coli"[Majr:NoExp]) OR "Gardner Syndrome"[Mesh:NoExp]) OR "Gardner Syndrome"[Majr:NoExp]) OR "Intestinal Polyps"[Mesh:NoExp]) OR "Intestinal Polyps"[Majr:NoExp]) OR "Colonic Polyps"[Mesh:NoExp]) OR "Colonic Polyps"[Majr:NoExp]) OR "Colorectal Neoplasms, Hereditary Nonpolyposis"[Mesh:NoExp]) OR "Colorectal Neoplasms, Hereditary Nonpolyposis"[Majr:NoExp]) OR "Rectal Neoplasms"[Mesh:NoExp]) OR "Rectal Neoplasms"[Majr:NoExp]) OR (“colorectal neoplas*” [tw] OR “epithelial poly*” [tw] OR “adenomatous poly*” [tw] OR “polyposis col* adenomatous” [tw] OR “polyposis syndrome” [tw] OR “hereditary polypos*, familial” [tw] OR “myh-associated polypos*” [tw] OR “sessile serrated poly*” [tw] OR “colorectal cance*” [tw] OR “recurrent colorectal cance*” [tw] OR “colorectal cancer progression free survival” [tw] OR “colon* cance*” [tw] OR “cancer of the colon” [tw] OR “overall colorectal cancer survival” [tw] OR “colorectal tumo*” [tw] OR “carcinoma* colorectal” [tw] OR “colonic neoplas*” [tw] OR “colon* poly*” [tw] OR “rectal neoplas*” [tw] OR “gardner* syndrom*” [tw] OR “intestinal poly*” [tw] OR “sigmoid neoplas*” [tw] OR “sigmoid cance*” [tw])

AND

("Probiotics"[MeSH Terms:noexp] OR "Probiotics"[MeSH Major Topic:noexp] OR "Lactobacilli"[Text Word] OR "bifidobacterial"[Text Word]) OR ("Prebiotics"[MeSH Terms:noexp] OR "Prebiotics"[MeSH Major Topic:noexp] OR ("Inulin"[Text Word] OR "Fructooligosaccharides"[Text Word] OR "FOS"[Text Word] OR "galactooligosaccharides"[Text Word] OR "GtOS"[Text Word] OR "xylooligosaccharides"[Text Word] OR "XOS"[Text Word] OR "isomaltooligosaccharides"[Text Word] OR "IMO"[Text Word] OR "beta-glucans"[Text Word] OR

"polyphenols"[Text Word] OR "lactulose"[Text Word])) OR ("microbiome"[Text Word] OR "microbiota"[Text Word]) OR ("yogurt"[Text Word])

AND

("randomized controlled trial"[Publication Type] OR "controlled clinical trial"[Publication Type] OR "randomized"[Title/Abstract] OR "placebo"[Title/Abstract] OR "drug therapy"[MeSH Subheading] OR "randomly"[Title/Abstract] OR "observational"[Title/Abstract] OR "cohort"[Title/Abstract] OR "case control*"[Title/Abstract] OR "cross-sectional"[Title/Abstract] OR "cross-sectional"[Title/Abstract] OR "trial"[Title/Abstract] OR "groups"[Title/Abstract]) NOT ("animals"[MeSH Terms] NOT "humans"[MeSH Terms])

Embase – Search performed 30 April, 1200 results // Search performed 12 May, 1204 results

**#1**	('colorectal neoplas*,':ab,ti OR 'epithelial poly*,':ab,ti OR 'adenomatous poly*,':ab,ti OR 'polyposis col*,':ab,ti OR 'adenomatous,':ab,ti OR 'polyposis syndrome,':ab,ti OR 'hereditary polypos*,':ab,ti OR 'familial,':ab,ti OR 'myh-associated polypos*,':ab,ti OR 'sessile serrated poly*,':ab,ti OR 'colorectal cance*,':ab,ti OR 'recurrent colorectal cance*,':ab,ti OR 'colorectal cancer progression free survival,':ab,ti OR 'colon* cance*,':ab,ti OR 'cancer of the colon,':ab,ti OR 'overall colorectal cancer survival,':ab,ti OR 'colorectal tumo*,':ab,ti OR 'carcinoma* colorectal,':ab,ti OR 'colonic neoplas*,':ab,ti OR 'colon* poly*,':ab,ti OR 'rectal neoplas*,':ab,ti OR 'gardner* syndrom*,':ab,ti OR 'intestinal poly*,':ab,ti OR 'sigmoid neoplas*,':ab,ti OR 'sigmoid cance*':ab,ti OR 'colorectal tumor'/mj OR 'colorectal adenoma'/exp/mj OR 'colon tumor'/mj OR 'colon adenoma'/mj OR 'colon polyp'/mj OR 'colon polyposis'/mj OR 'gardner syndrome'/mj OR 'intestine polyp'/mj OR 'rectum tumor'/mj)
**#2**	('inulin,':ab,ti OR 'fructose oligosaccharide,':ab,ti OR 'fructooligosaccharides,':ab,ti OR 'fos,':ab,ti OR 'galactose oligosaccharide':ab,ti OR 'galactooligosaccharides':ab,ti OR 'gtos':ab,ti OR 'xylooligosaccharides':ab,ti OR 'xos':ab,ti OR 'isomaltooligosaccharide':ab,ti OR 'polyphenol':ab,ti OR 'lactulose':ab,ti OR 'β-glucans':ab,ti OR 'prebiotic agent'/mj OR 'lactobacillus':ab,ti OR 'bifidobacteriales':ab,ti OR 'probiotic agent'/mj OR ‘yogurt’)

**#3**	('case control study'/de OR 'clinical study'/de OR 'clinical trial'/de OR 'clinical trial topic'/de OR 'comparative study'/de OR 'controlled clinical trial'/de OR 'controlled study'/de OR 'human'/de OR 'intervention study'/de OR 'observational study'/de OR 'practice guideline'/de OR 'prospective study'/de OR 'randomized controlled trial'/de OR 'randomized controlled trial topic'/de)
**#4**	‘human’/de
**#5**	#1 AND #2 AND #3 AND #4

Web of Science – search completed 30 April 2021, 1188 results // 12 May, 1236 results

- *Indexes=SCI-EXPANDED, SSCI, A&HCI, CPCI-S, CPCI-SSH, BKCI-S, BKCI-SSH, ESCI, CCR-EXPANDED*
- **Document Types: Article**

**#1**	(TS=("Inulin" OR "Fructooligosaccharides" OR "FOS" OR "galactooligosaccharides" OR "GtOS" OR "xylooligosaccharides" OR "XOS" OR "isomaltooligosaccharides" OR "IMO" OR "β-glucans" OR "polyphenols" OR "lactulose" OR "prebiotic*" OR "prebiotic interventions"))
**#2**	(TS=("Lactobacilli" OR "bifidobacterial" OR "probiotic*" OR “yogurt”))
**#3**	(TS=("synbiotic*"))
**#4**	(TS=("colorectal neoplas*" OR "epithelial poly*" OR "adenomatous poly*" OR "polyposis col*, adenomatous" OR "polyposis syndrome" OR "hereditary polypos* familial" OR "myh-associated polypos*" OR "sessile serrated poly*" OR "colorectal cance*" OR "recurrent colorectal cance*" OR "colorectal cancer progression free survival" OR "colon* cance*" OR "cancer of the colon" OR "overall colorectal cancer survival" OR "colorectal tumo*" OR "carcinoma* colorectal" OR "colonic neoplas*" OR "colon* poly*" OR "rectal neoplas*" OR "gardner* syndrom*" OR "intestinal poly*" OR "sigmoid neoplas*" OR "sigmoid cance*" OR "rectal neoplas*"))
**#5**	(TS= ((randomized controlled trial OR controlled clinical trial OR randomized OR placebo OR randomly OR observational OR cohort OR “case-control” OR “case control” OR “cross-sectional” or “cross sectional” OR trial OR groups OR human*)<br>NOT (animals NOT humans)))

**#6**	#1 or #2 or #3
**#7**	#4 and #6
**#8**	#7 and #5

Cochrane Reviews & Trials – Search performed 30 April 2021

1 review, 62 trials //12 May search - 1 review, 62 trials

**#1**	("colorectal neoplas*" OR "epithelial poly*" OR "adenomatous poly*" OR "polyposis col*, adenomatous" OR "polyposis syndrome" OR "hereditary polypos* familial" OR "myh-associated polypos*" OR "sessile serrated poly*" OR "colorectal cance*" OR "recurrent colorectal cance*" OR "colorectal cancer progression free survival" OR "colon* cance*" OR "cancer of the colon" OR "overall colorectal cancer survival" OR "colorectal tumo*" OR "carcinoma* colorectal" OR "colonic neoplas*" OR "colon* poly*" OR "rectal neoplas*" OR "gardner* syndrom*" OR "intestinal poly*" OR "sigmoid neoplas*" OR "sigmoid cance*" OR "rectal neoplas*" OR "colo-rectal")
**#2**	MeSH descriptor: [Colorectal Neoplasms] this term only
**#3**	MeSH descriptor: [Sigmoid Neoplasms] this term only
**#4**	MeSH descriptor: [Adenomatous Polyposis Coli] this term only
**#5**	MeSH descriptor: [Gardner Syndrome] this term only
**#6**	MeSH descriptor: [Intestinal Polyps] this term only
**#7**	MeSH descriptor: [Colonic Polyps] this term only
**#8**	MeSH descriptor: [Colorectal Neoplasms, Hereditary Nonpolyposis] this term only
**#9**	MeSH descriptor: [Rectal Neoplasms] this term only
**#10**	(“Inulin” OR “Fructooligosaccharides” OR “FOS” OR “galactooligosaccharides” OR “GtOS” OR “xylooligosaccharides” OR “XOS” OR “isomaltooligosaccharides” OR “IMO” OR “β-glucans” OR “polyphenols” OR “lactulose” OR "prebiotic*" OR "prebiotic interventions") OR (“Lactobacilli” OR “bifidobacterial” OR "probiotic*") OR ("synbiotic*") OR (“yogurt”),

**#11**	MeSH descriptor: [Probiotics] this term only
**#12**	MeSH descriptor: [Prebiotics] this term only
**#13**	#1 or #2 or #3 or #4 or #5 or #6 or #7 or #8 or #9
**#14**	#10 or #11 or #12
**#15**	#13 and #14

Clinical Trials

("colorectal neoplas*" OR "epithelial poly*" OR "adenomatous poly*" OR "polyposis col*, adenomatous" OR "polyposis syndrome" OR "hereditary polypos* familial" OR "myh-associated polypos*" OR "sessile serrated poly*" OR "colorectal cance*")
